# News and Events of the Week

**Published:** 1910-12-24

**Authors:** 


### News and Events of the Week.

An election of a member of the Court of Examiners will take place at the next meeting of the Council of the Royal College of Surgeons. Fellows of the college desirous of becoming candidates for the office should make application in writing to the Secretary on or before January 4.

The new slaughter-houses opened at Islington last week are extensions of the public slaughter-houses recently built by the Corporation, and have been erected under the direction of the City Surveyor at a cost of £60,000. They are the first to be established in London. All condemned meat and offal is converted into a dry, soft powder, which finds a ready market as a fertiliser.

A glance at the list of prizes annually awarded by the French Academy of Medicine reveals the gratifying fact that a large number of these awards have been assigned to private practitioners. In England the private practitioner does not trouble himself much about medical research, but his French colleague is fully aware of the importance of even minor clinical observations, and the articles and treatises which have been awarded prizes are really evidences of good work achieved in a humble way.

Mr. R. J. Pye-Smith has resigned the Professorship of Surgery at the University of Sheffield. The Council accepted the resignation with great regret, and passed a resolution recording its appreciation of his services to the cause of medical education in Sheffield. Mr. Pye-Smith, who is the senior member of the teaching staff, has been a teacher of surgery for thirty-four years, first in the old Medical School, next in the University College, and finally in the University.

Among the handy reference books which reach us every year at Christmas time Messrs. Adam and Charles Black's universally popular handbooks, "Who's Who," "The Writers' and Artists' Year Book," and "The Englishwoman's Year Book," deserve special mention. They are not exhaustive, it is true—"Who's Who" contains, so far as the medical profession is concerned, the names of many nonentities and omits all reference to doctors who have a deservedly high reputation—but they are interesting and useful. For that reason their annual appearance is very welcome, and those who do not already know them should make a point of writing to Messrs. Black—whose address is Soho Square, London—for particulars about these reference annuals.

The London Education Committee received last week from the Children's Care Sub-Committee a letter from the Board of Education, dealing with the arrangements for the medical inspection of children attending public elementary schools. The Board states that investigations have disclosed a great lack of that organisation, direction, and control of the medical staff which is essential in dealing with the enormous area and numbers involved in the case of London schools, and particularly when the work is attempted by means of a number of part-time school doctors. The Board cannot find any clear indication of the means by which the Council are remedying or are proposing to remedy these defects. With regard to the dental treatment of children, a letter from the Board of Education states that there is an obvious need that early action should be taken to deal with defects which seriously threaten the children's physical welfare.

Mr. Cecil Huntington Leaf, F.R.C.S., of Wimpole Street, W., who died on October 5, left estate of the gross value of £20,682, with net personalty £20,392.

The annual report of Livingstone College, where missionary medical men are specially trained, has just been issued. The great need of the College at the present moment is to obtain a regular supply of students, but financial help is also necessary to remove the deficiency of £275,000.

The 1911 edition of "Whitaker's Almanack" is as good as ever, and it could not be much better than it was last year. It is no use quoting from such a compendious volume as "Whitaker," but, as a sample of its up-to-dateness, we may mention that the pertinent facts concerning the Crippen case are contained in it.

Mr. Thomas Wright, of Castle Place, Nottingham, consulting surgeon to the Nottingham General Hospital, formerly Surgeon-Lieutenant-Colonel of the Robin Hood Rifles, at one time a well-known shot and angler, who died on October 25, aged eighty-eight, has left estate valued at £30,371 gross and £30,309 net.

The Lord Lieutenant of Ireland and the Countess of Aberdeen left Paris on Tuesday for Lille to make a further inspection of sanatoria for consumptives in the Department of the Nord. Their Excellencies were accompanied by Sir William Thompson, consulting physician to the National Hospital for Consumption in Ireland.

In view of the interest excited in our columns by the recent correspondence between the King's Fund and its critics, Sir John Wolfe-Barry's remarks on the relation of the hospital to medical school, which he made at the presentation of prizes to the Westminster Hospital students last week, are worth quoting: "The House Committee," he said, "and the governors recognised the immense advantage of an alliance between the hospital and the school. The school looked forward to a close alliance with the Board of Education, which was going to help them greatly, and the Board was to be represented on the governing body. The House Committee had viewed with great interest the advance made by vaccine-therapy, and had started a laboratory for the investigation of the subject." Lord Ilkeston, who presented the prizes, looked forward with some apprehension to the prospects of the general practitioner, who after all, he said, was the backbone of the profession. Students of Westminster Hospital were also students of the London School of Medicine. That school was probably the worst organised in the world, but, nevertheless, it was the greatest and in some ways the best school, for it contained such a vast wealth of clinical material. On that account he besought them to keep the medical school and the hospital closely allied. London would be all the better for more concentration in scientific teaching. These latter remarks are a striking illustration of our first leading article in The Hospital of December 17.

Messrs. Black will shortly publish in the Menpes series of pictures a reproduction in colour of Mr. Hugh de T. Glazebrook's portrait of King George V., which represents his Majesty in uniform as Colonel-in-Chief of the Royal Marine Artillery.

